# The clinical characteristics and risk factors for necrotizing pneumonia caused by *Mycoplasma pneumoniae* in children

**DOI:** 10.1186/s12879-020-05110-7

**Published:** 2020-06-01

**Authors:** Baoying Zheng, Jing Zhao, Ling Cao

**Affiliations:** Department of Pulmonology, Capital Institute of Pediatrics-Peking University Teaching Hospital, Beijing, China

**Keywords:** Necrotizing pneumonia, *Mycoplasma pneumoniae*, Children, Risk factor

## Abstract

**Background:**

The incidence of necrotizing pneumonia (NP) caused by *Mycoplasma pneumoniae* (MP) is increasing. We analyzed the clinical characteristics and the risk factors for NP caused by MP.

**Methods:**

A retrospective observational study was conducted in 37 patients with NP caused by MP (NP group) and 74 patients diagnosed with lobar *M. pneumoniae* pneumonia with no necrosis (control group) who were admitted to our hospital between January 2013 and December 2017. The clinical manifestations, laboratory data, imaging findings, treatments and outcomes were analyzed.

**Results:**

The proportion of females, the incidence of pleural effusion, fever duration, hospitalization days, white blood cell count, neutrophil ratio, D-dimer level and use of other types of antibiotics were higher in the NP group than in the control group (*P* < 0.05). The control group exhibited a greater use of low molecular weight heparin (LMWH) than the NP group (*P* < 0.05). According to the multivariate logistic regression analysis, a white blood cell count > 12.3 × 10^9^/L (Odds ratio, OR = 6.412), a neutrophil ratio > 73.9% (OR = 6.081) and D-dimer level > 1367.5 ng/mL (OR = 8.501) were risk factors for pulmonary necrosis caused by MP. Furthermore, the use of LMWH (OR = 0.074) reduced the risk of pulmonary necrosis.

**Conclusions:**

NP is a rare complication of severe *Mycoplasma pneumoniae* pneumonia (SMPP), and although the clinical course is longer than common MP infection, the necrotic area is absorbed gradually. In patients with SMPP presenting with lobar consolidation, a white blood cell count > 12.3 × 10^9^/L, a neutrophil ratio > 73.9% and D-dimer level > 1367.5 ng/mL are risk factors for pulmonary necrosis, and the use of LMWH reduces the risk of pulmonary necrosis.

## Background

*Mycoplasma pneumoniae* (MP) is one of the most important pathogens responsible for community-acquired pneumonia in school-aged children and young adults [1]. The clinical course of *M. pneumoniae* pneumonia (MPP) is typically mild and self-limiting [2], but severe *Mycoplasma pneumoniae* pneumonia (SMPP) has been reported, and a number of severe pulmonary complications, including obliterative bronchitis, bronchiectasis and necrotizing pneumonia (NP), have been reported [3].

NP is a rare manifestation of MP infection [4], and the literature focused on NP caused by *M. pneumoniae* infection is scarce; no study has analyzed the risk factors for pulmonary necrosis. In the present study, we reviewed the records of 37 patients with NP caused by MP who were admitted to our hospital and compared them with 74 patients who were diagnosed with lobar MPP without necrosis. We analyzed the clinical characteristics of these patients and the risk factors for NP caused by MP.

## Methods

### Patients

Thirty-seven patients with NP caused by MP (NP group) who were admitted to The Affiliated Children’s Hospital of Capital Institute of Pediatrics from January 1, 2013, to December 31, 2017, were identified. Seventy-four patients diagnosed with lobar MPP without necrosis (control group) were also included. This study was approved by the Ethics Committee of Capital Institute of Pediatrics-Peking University Teaching Hospital (no. SHERLL2019061).

### Diagnostic criteria

A diagnosis of pneumonia was defined as follows [5]: acute respiratory infection symptoms (fever, cough or wheezing) upon a physical examination and chest imaging with infiltrates. Severe pneumonia was defined as pneumonia plus one of the following characteristics [6]: (1) a poor general condition, (2) an increased respiratory rate (infants > 70 breaths/min and older children > 50 breaths/min), (3) dyspnea and cyanosis, (4) multilobe involvement or involvement of ≥2/3 of the lung, (5) extrapulmonary complications, (6) pleural effusion, and (7) transcutaneous oxygen saturation in room air ≤92%.

The MP infection was confirmed by conducting a serological test (MP-IgM-positive and an antibody titer ≥1:160 or a four-fold or greater increase in the titer) and MP nucleic acid detection from the nasopharyngeal aspirate and BAL and/or pleural effusion [7, 8]. In our study, the antibody titers were ≥ 1:640 in both the NP group and control group. In addition, 55 (49.5%) patients also tested for nucleic acids of *M. pneumoniae* in nasopharyngeal or bronchoalveolar lavage fluid, and the results were positive. SMPP was defined as severe pneumonia with an MP infection.

The diagnosis of NP mainly depends on chest imaging. In plain chest radiographs, necrotic lesions may present as density-reduced areas and cavity lesions. Chest computed tomography (CT) manifestations of NP were the destruction of normal lung parenchymal structures and a decrease in parenchymal enhancement with structures that were gradually replaced by multiple small air- or fluid-filled cavities with thin, nonenhanced walls [9–11].

### Inclusion and exclusion criteria

Because all patients in the NP group and control group were diagnosed with SMPP, a chest CT was performed on patients in both groups while they were in the hospital. Therefore, the diagnosis of NP was based on the CT manifestations. Forty-three (38.7%) patients underwent a contrast-enhanced chest CT.

The NP group included patients who were diagnosed with community-acquired pneumonia caused by MP and in whom necrotic lesions were observed on the chest CT. All patients presented with lobar consolidation early in the course of the disease. Cultures for bacteria and fungus were negative in all patients. Bacterial nucleic acids were not detected.

For the control group, we selected children diagnosed with SMPP with lobar consolidation and in whom the lung lesions gradually improved. No necrotic lesions were observed within 3 months. Cultures for bacteria and fungus were negative. Bacterial nucleic acids were not detected. One hundred eighty-four patients met these conditions to match the NP group and had complete clinical data available. The patients were numbered, and 74 patients were randomly selected using the random number Table. A statistically significant difference in age was not observed between the two groups (*P* > 0.05).

Children who presented an immunocompromised state or with chronic lung diseases were excluded from this study. We also excluded children with other cavitary diseases, such as lung abscess, pulmonary cyst with infection, and pulmonary tuberculosis. In addition, patients with a family history of embolism were excluded.

### Data collection

Clinical information was retrospectively collected from the medical records of the patients. The age, sex, clinical symptoms and signs, intrapulmonary and extrapulmonary complications, fever duration, hospitalization days and treatments were recorded. The maximum levels of the laboratory tests observed during hospitalization, including white blood cell (WBC) counts, neutrophil counts, and levels of C-reactive protein (CRP), procalcitonin (PCT), lactate dehydrogenase (LDH), and D-dimer, were recorded. Blood, pleural effusion and nasopharyngeal aspirate/bronchoalveolar lavage fluid cultures, virus antigen detection assays (respiratory syncytial viruses, adenovirus, metapneumovirus, influenza, and parainfluenza), interferon-γ release assays and T cell spot tests (T-SPOTs) for a tuberculosis infection were performed. Thirty-five (31.5%) patients underwent bacterial nucleic acid detection (*Acinetobacter baumannii, Escherichia coli, Klebsiella pneumoniae, Streptococcus pneumoniae, Chlamydia pneumoniae, Staphylococcus aureus, Haemophilus influenzae*, methicillin-resistant *Staphylococcus*, *Legionella pneumophila*, *Pseudomonas aeruginosa, Pseudomonas maltophilia, Mycobacterium tuberculosis*). Fifty-five (49.5%) patients were tested for nucleic acids of *M. pneumoniae*. A chest CT was performed during hospitalization. Repeated imaging examinations, including plain chest radiographs and chest CT, were performed within the follow-up period. Some children underwent fiber bronchoscopy with bronchoalveolar lavage. An electrocardiogram, echocardiography and abdominal ultrasound were performed in all patients.

### Statistical analysis

The statistical analyses were performed using SPSS software (IBM, version 22). Normally distributed data are reported as means±standard deviations (SDs). Data with a skewed distribution are presented as median values (interquartile ranges: Q1, Q3). The different groups were compared using a two-sample t test or the Mann-Whitney U test. The statistical significance of the differences in the categorical variables was determined using the chi-square test and Fisher’s exact test. Statistical significance was determined at the two-tailed 0.05 level. A multivariate logistic regression analysis was performed to identify the risk factors for NP caused by MP. Some continuous variables, such as the fever duration, WBC counts, neutrophil ratio, CRP levels and D-dimer levels, were categorized into the 30th percentile, 60th percentile and 90th percentile.

## Results

### Clinical features (Tables 1 and 2)

All patients presented with a fever and cough. The total fever duration was 18.3 ± 8.7 days in NP group and 11.4 ± 2.9 days in the control group. Three patients (8.1%) in the NP group suffered from chest pain, and none of the patients in the control group presented with chest pain. Four patients (10.8%) in the NP group and 3 patients (4.1%) in the control group suffered from dyspnea. Twenty-three patients (62.2%) in the NP group and 29 patients (39.2%) in the control group had pleural effusion. Three patients (8.1%) in the NP group and 9 patients (12.2%) in the control group experienced atelectasis. Sixteen patients (43.2%) in the NP group and 38 patients (51.4%) in the control group presented with extrapulmonary manifestations, of which abnormal liver function was the most common symptom.

**Table 1:** Clinical information and treatment of the two groups

Clinical information and treatment	NP group (*n* = 37)	Control group (*n* = 74)	*P* value
Clinical presentation (n, %)			
Fever	37, 100%	74, 100%	–
Cough	37, 100%	74, 100%	–
Chest pain	3, 8.1%	0	0.035^a^
dyspnea	4, 10.8%	3, 4.1%	0.334
Extrapulmonary complications (n, %)			
Elevated liver enzyme level	10, 27%	28, 37.8%	0.258
Electrolyte disturbance	4, 10.8%	6, 8.1%	0.907
Hypoproteinemia	4, 10.8%	2, 2.7%	0.182
Coronary dilatation	0	1, 1.4%	1.000^a^
Pericardial effusion	0	1, 1.4%	1.000^a^
Polymorphic erythema	0	1, 1.4%	1.000^a^
Treatment (n, %)			
Administered other antibiotics	34, 91.9%	51, 68.9%	0.007
Noninvasive ventilation	1, 2.7%	0	0.722
Low molecular weight heparin	13, 35.1%	42, 58.1%	0.032
Methylprednisolone	30, 81.1%	65, 87.8%	0.339
Intravenous immunoglobulin	10, 27%	8, 10.8%	0.029
Fiber bronchoscope examination	23, 62.2%	48, 64.9%	0.780
Mucous congestion, edema	23, 100%	48, 100%	0.780
Bronchitis obliterans	1, 4.3%	0	0.324^a^
Plastic bronchitis	1, 4.3%	0	0.324^a^

^a^ Fisher’s exact test

**Table 2:** Comparison of the clinical indexes

Clinical index	NP group (*n* = 37)	Control group (*n* = 74)	*P* value
Female (n, %)	28, 75.7%	41, 55.4%	0.038
Age (years)	6.7 ± 2.5	7.1 ± 2.3	0.402
Total fever duration (days)	18.3 ± 8.7	11.4 ± 2.9	< 0.001
Pleural effusion (n, %)	23, 62.2%	29, 39.2%	0.027
Atelectasis (n, %)	3, 8.1%	9, 12.2%	0.748
Extrapulmonary Complications (n, %)	16, 43.2%	38, 51.4%	0.197

Data are presented as means ± SDs

Significant differences in age, the incidence of atelectasis and extrapulmonary complications were not observed between the two groups (*P* > 0.05). The NP group included a higher proportion of females than the control group (*P* < 0.05). The total fever duration and the incidence of chest pain and pleural effusion in the NP group were higher than in the control group (*P* < 0.05).

### Laboratory data

A portion of the laboratory data are presented in Table 3. A significant difference in LDH levels was not observed between the two groups (*P* > 0.05). Higher CRP levels, WBC counts, neutrophil ratios, and D-dimer levels were observed in the NP group than in the control group (*P* < 0.05).

**Table 3:** Comparisons of laboratory data

Laboratory data	NP group (*n* = 37)	control group (*n* = 74)	*P* value
WBC count (×10^9^/L)	13.28 (9.74, 15.92)	10.05 (8.4, 11.98)	< 0.001
Neutrophil (%)	74.87 ± 8.34	67.46 ± 9.11	< 0.001
CRP (mg/L)	62.7 (32.9, 170)	38.67 (20.98, 85.6)	0.010
LDH (U/L)	524 (329, 616)	436.5 (341.75, 522.5)	0.215
D-dimer (ng/mL)	1411 (930.9, 3363.5)	859 (469.5, 1333.75)	< 0.001

Data are presented as means±SDs or medians (Q1, Q3)

In the NP group, PCT levels were measured in 32 patients; 25 (78.1%) patients presented normal PCT levels (< 0.5 ng/mL), but only 2 (6.3%) presented PCT levels > 10 ng/mL. In the control group, PCT levels were measured in 70 patients; 58 (82.9%) presented normal PCT levels and no patient presented a PCT level > 10 ng/mL. A statistically significant difference in the proportion of patients with increased PCT levels was not observed between the two groups (*P* > 0.05).

In the NP group, two patients were coinfected with respiratory syncytial viruses, and one patient was coinfected with adenovirus. In the control group, one patient was coinfected with adenovirus, and one was coinfected with parainfluenza virus.

### Radiological findings

In the NP group, chest CT showed lung consolidation in the early stage of the disease; 32 patients presented unilateral lung consolidation, with one or more affected lobes. Decreased enhancement areas were observed in 17 patients on the contrast-enhanced chest CT images, and pulmonary cavities were observed in 22 patients. The mean time from the onset of symptoms to the discovery of necrotic lesions was 24.92 ± 10.84 days. Changes in CT signs were recorded in 12 patients during follow-up. Eight patients underwent a chest CT examination at 2–3 months after the onset of disease. Pulmonary consolidation was observed in 4 patients. One patient presented cavities in the lung, 1 presented with atelectasis and 1 presented with pleural thickness. A chest CT was performed after 6 months in 3 patients, with 1 presenting cavities in the lung. Two patients underwent a chest CT examination after 1 year, one had atelectasis and bronchiectasis, and one had fibrous stripes in the lung.

### Treatments and outcomes

Because quinolones are not approved for use in children under 18 and tetracycline is not approved for use in children under 8 by the China Food and Drug Administration (CFDA), we treated the MP infection with macrolides. Many patients, even up to 91.9% in the NP group, were administered other antibiotics in addition to macrolides, such as a cephalosporin, vancomycin, or imipenem, because of the severity of the disease and the significantly increased WBC counts and CRP and PCT levels. Methylprednisolone (1–2 mg/kg, 2–3 times a day) and intravenous immunoglobulin were administered to some patients with an acute onset, rapid progression and serious illness, namely, 81.8 and 27% in the NP group and 87.8 and 10.8% in the control group, respectively. In out hospital, low molecular weight heparin (LMWH) was administered at a dose of 50–100 mg/kg, 1–2 times a day when D-dimer levels increased. In the present study, some children had been treated in other hospitals for a period of time before arriving at our hospital, and LMWH was not applied before the necrotic lesion appeared, even if the D-dimer levels increased. Thirteen patients (35.1%) in the NP group and 42 patients (58.1%) in the control group were treated with LMWH (Table 1).

Significant differences in the use of LMWH and other antibiotics were observed. The use of LMWH in the control group was greater than in the NP group (*P* < 0.05), and the use of other antibiotics and intravenous immunoglobulin in the NP group was greater than in the control group (*P* < 0.05). The median number of hospitalization days was 22 days in the NP group and 11 days in the control group. The NP group was hospitalized for more days than the control group (*P* < 0.05).

### Multivariate logistic regression analysis

According to the multivariate logistic regression analysis (Table 4), a WBC count > 12.3 × 10^9^/L (OR = 6.412), a neutrophil ratio > 73.9% (OR = 6.081) and D-dimer level > 1367 ng/mL (OR = 8.501) were risk factors for pulmonary necrosis caused by MP. Furthermore, the use of LMWH (OR = 0.074) reduced the risk of pulmonary necrosis.

**Table 4:** Results of the multivariate logistic regression analysis

Parameter	OR	95% CI	*P* value
Fever duration (< 10 days)	1		
10–14 days	0.559	0.115–2.725	0.472
> 14 days	4.719	0.943–23.608	0.059
WBC count (< 9.5 × 10^9^/L)	1		
9.5 × 10^9^–12.3 × 10^9^/L	1.886	0.42–8.476	0.408
> 12.3 × 10^9^/L	6.412	1.483–27.728	0.013
Neutrophil ratio (< 66.8%)	1		
66.8–73.9%	1.751	0.395–7.767	0.461
> 73.9%	6.081	1.149–32.19	0.034
CRP (< 32 mg/L)	1		
32–82 mg/L	0.777	0.158–3.819	0.757
> 32 mg/L	0.956	0.158–5.781	0.961
D-dimer (< 635.8 ng/mL)	1		
635.8–1367.5 ng/mL	1.999	0.158–5.781	0.388
> 1367.5 ng/mL	8.501	1.282–56.385	0.027
Female	3.734	0.94–14.836	0.061
Pleural effusion	2.632	0.82–8.45	0.104
Use of LMWH	0.074	0.015–0.36	0.001

## Discussion

MP is considered an important pathogen that causes community-acquired pneumonia in children [3, 12]. In China, SMPP is becoming increasingly common, and is characterized by a rapid onset, rapid progression, severe pulmonary lesions, and prolonged course, with a persistent high fever and severe cough as the clinical symptoms. Intrapulmonary and extrapulmonary complications are often associated with SMPP. A number of severe pulmonary complications, including NP, have been reported.

NP is characterized by the necrosis and liquification of the lung tissues, which are replaced by cavities that are surrounded by walls of varying thicknesses [3, 13]. If not adequately treated, NP may lead to complications, including bronchopleural fistula, empyema, respiratory failure, and septic shock [14]. Bacterial infections, particularly infections of *Streptococcus pneumoniae* and *Staphylococcus aureus,* are the most common etiology [13, 15]. Since NP caused by MP infection was first reported by Oermann [16] in 1997, an increasing number of cases have been reported in the literature.

When the lungs are necrotic, a child often has no complaints of special discomfort. Lung necrosis can be detected at an early stage through contrast-enhanced chest CT, but it is undetectable in plain chest radiographs. Chest CT shows lung consolidation in the early stage of the disease, and decreased parenchymal enhancement is observed on contrast-enhanced chest CT. Finally, pulmonary cavities are observed. In the present study, the mean time from the onset of symptoms to the discovery of necrotic lesions was 24.92 ± 10.84 days. Previous studies [4, 9, 10, 17] have showed that the consolidation and necrotic area will be absorbed gradually, and the lung cavity disappears. Residual fibrous stripes, atelectasis, and lobar cystic degeneration have been observed in some patients within 3–6 months; furthermore, these signs may persist for 1–3.5 years. In our study, most of the patients lived in other provinces, and long-term follow-up was difficult to achieve. Follow-up showed the disappearance of most of the cavity lesions within 3–6 months, consistent with previous reports.

In patients with SMPP, local hypoxia, ischemia and acidosis occur. In addition, the direct invasion of pathogens and their toxins will activate the coagulation system and form a hypercoagulable state. At the same time, the fibrinolysis, kinin and complement systems in the body will also be activated successively, which may cause the abnormal coagulation function [18]. Thromboembolism due to MP infection has been reported in the literature [19]. As a specific marker of fibrinolysis, D-dimer reflects the capacity to dissolve fibrin, and increasing evidence suggests that coagulation function is closely related to the inflammatory response [20]. The clinical symptoms and the chest imaging findings of patients with MPP presenting with elevated D-dimer levels are more serious than patients with normal D-dimer levels [21, 22]. In our study, the NP group presented a noticeably higher D-dimer level than the control group, consistent with other reports [23]. Furthermore, a D-dimer level > 1367 ng/mL (OR = 8.501) was a risk factor for pulmonary necrosis. Therefore, when D-dimer levels increase significantly, we should pay attention to the possibility of pulmonary necrosis.

Currently, LMWH is consistently administered as a treatment for hypercoagulation. In addition to anticoagulant and antithrombotic functions, LMWH has many biological activities and pharmacological mechanisms, such as anti-inflammatory effects, anti-fibrosis activity, immune regulation and cell proliferation inhibition [24, 25]. As shown in the study by Zhang CF et al. [22], early use of LMWH accelerates the absorption of lung lesions, shortens the hospitalization time and improves the prognosis of children with SMPP. In our study, the multivariate logistic regression analysis showed that use of LMWH (OR = 0.074) reduced the risk of pulmonary necrosis, indicating that treatment with LMWH played an important role in SMPP.

According to the multivariate logistic regression analysis, a WBC count > 12.3 × 10^9^/L and a neutrophil ratio > 73.9% were also risk factors for pulmonary necrosis. However, WBC counts and neutrophil ratios are easily affected by other factors, such as coinfection with bacteria and the use of glucocorticoids. Therefore, their predictive effects must be analyzed together with other indicators.

The present study had several limitations. First, it was a retrospective observational study, and the patients enrolled were from a single center. Second, the sample size of the NP group was relatively small.

## Conclusions

In conclusion, NP is a rare complication of SMPP, and although the clinical course is longer than a common MP infection, the necrotic area is absorbed gradually. In patients with SMPP presenting with lobar consolidation, a WBC count > 12.3 × 10^9^/L, a neutrophil ratio > 73.9% and D-dimer level > 1367.5 ng/mL, we should be alert to the occurrence of pulmonary necrosis, and the use of LMWH reduced the risk of pulmonary necrosis.

## Data Availability

Datasets analyzed in this study can be obtained from the corresponding author upon reasonable request.

## Abbreviations

CFDA: China Food and Drug Administration
CRP: C-reactive protein
CT: computed tomography
LDH: lactate dehydrogenase
LMWH: low molecular weight heparin
MP: *Mycoplasma pneumoniae*
NP: necrotizing pneumonia
PCT: procalcitonin
SDs: standard deviations
SMPP: severe *Mycoplasma pneumoniae* pneumonia
T-SPOTs: T cell spot tests
WBC: white blood cell
